# The Idea of Wealth

**Published:** 1873-11

**Authors:** 


### THE IDEA OF WEALTH.

It is said that John Jacob Astor, who never was worth half as much as his son now is, advised a man who had made a good start in life, not to try to be *wealthy*, adding: "It's only a vexation; a man who has four or five hundred thousand dollars is as well off as if he were rich." The millionaire was right, or if he erred, it was in the hight of his estimate. We can use a little money for our personal needs—not much; and as to the rest, we can give away a little—not a great deal, if we give it wisely. After that, the remainder is a burden—a sheer load, which wears a man out in the care of it. Suppose the case of a man worth between forty and fifty millions of dollars, and there are not a few such in the world. He would receive on this property, if it were made productive, a round income of five per cent, or $2,250,000 per annum. Now we have no clearer idea of two millions of dollars than we have of forty millions. It is a great deal of money; we must reduce it again, to make it manageable.

We will suppose that the income of the reader is $2400 per annum, which is much above the average. If he receives it regularly at the end of his day's work, it will be six dollars and sixty-seven cents per diem—a sufficient sum, on which a large family may be supported and children educated, with something to spare for the poor. The difference between this amply-provided person and Mr. Astor, is, that the latter receives something more than $6000 every day.

If the six dollar man thinks he works any harder than he of the six thousand, he is much mistaken; if he thinks that the other enjoys more substantial comfort, he is equally in error.

We should have a great deal of consideration and sympathy for rich men. Their roses have thorns, and are finer to look at than to have in the hand. The owner of the shining parterre is, after all, not as well off as the careless passer-by, who enjoys all its beauties without one of its responsibilities. There is the responsibility of benevolence, for example. Every body says that a rich man ought to give away a great deal of money. This requires no small degree of good judgment. A wide and indiscriminate almsgiving does no good; on the contrary, much harm. The laws of God and nature as to private thrift are identical. There is a niggardliness, to be sure, which is easily strengthened in the human heart, and which turns it, at length, into a parchment money-purse. But no man's heart needs to fall into this shriveled condition. There are safe outlets for money enough to save the thriftiest of men from such a drying up. Even Mr. Crœsus Bags, burdened as he is by involuntary accumulations, could find the means of saving himself. But the notion that a man ought to give away every thing that he gains, beyond the comfortable support of himself and his immediate dependencies, is a hurtful fallacy. A man who is in health, is not morally entitled to any thing which he does not earn. And, as all moral laws are found to be consistent with each other, it is ascertained that the acquisition of unearned money is almost uniformly a damage rather than a benefit. The moment a man is thus endowed, nature struggles to restore her standard of equilibrium by dispossessing him, and reducing him to his former position. The brief stay of inherited possessions, in the absence of legal protection for the mere inheritance of fraudulent gains, and of wasteful charities is the evidence. There are plentiful instances of real need, of cases in which palliative help is necessary, and in which it may not be rightly withheld. Those who are ready to perish from want; those who are in circumstances of spiritual destitution; those who are earnestly helping themselves against odds; those who are unwilling to receive alms, and can scarcely be induced to take it—are to be found every where, and afford an ample opportunity to deplete dangerous wealth. Nor should we *always* turn away from the whining mendicant, whether he come in cravat and broadcloth or in rags; sometimes even he may have a claim upon us. We should hear and judge.

Wealth belongs to some men, just as intellect belongs to others. They would be rich any where, just as their envious neighbors would be poor any where. At an agrarian meeting in this city about twenty years ago, a gentleman of property obtained a hearing and forcibly argued this point. Addressing a sailor near him, who had been prominent in the proceedings, he asked:

"What would you have me do with my money?"

"Divide it equally among us all," replied Jack.

"That would give us about $10 each, and to-morrow I should have $9.50 of mine left, while yours would be gone. What then?"

"Shiver my timbers!" exclaimed the sailor, in perplexity, "why—then divide again!"

